# Arterial resection and reconstruction in pancreatectomy: surgical technique and outcomes

**DOI:** 10.1186/s12893-019-0560-2

**Published:** 2019-10-10

**Authors:** Qiyi Zhang, Jingjin Wu, Yang Tian, Jixuan Duan, Yi Shao, Sheng Yan, Weilin Wang

**Affiliations:** 1 Department of Hepatobiliary and Pancreatic Surgery, The Second Affiliated Hospital, School of Medicine, Zhejiang University, Hangzhou, 310003 Zhejiang People’s Republic of China; 2 Division of Hepatobiliary and Pancreatic Surgery, Department of Surgery, The First Affiliated Hospital, Zhejiang University School of Medicine, Hangzhou, China; 3 General Surgery, The Fourth Affiliated Hospital, Zhejiang University School of Medicine, Yiwu, China

## Abstract

**Background:**

The outcomes in patients with pancreatic or ampulla tumors remain unsatisfactory, especially with invasion into the hepatic artery (HA) or the superior mesenteric artery (SMA). In this setting, pancreatectomy combined with arterial resection and reconstruction may offer the possibility of an en-block resection with negative margins and acceptable morbidity and mortality.

**Methods:**

A six year retrospective review of pancreatectomies performed at our institution, included 21 patients that underwent a pancreatectomy combined with arterial resection and reconstruction. Arterial reconstruction was performed under an operating microscope. The types of arterial reconstruction included direct anastomosis, arterial transposition, and arterial bypass with a vascular graft.

**Results:**

The surgical procedures consisted of 19 pancreaticoduodenectomies and 2 total pancreatectomies. The tumors were located at the pancreatic head (*n* = 10), whole pancreas (*n* = 2), distal common bile duct (*n* = 5), ampulla (*n* = 2) and retroperitoneum with pancreatic head involvement (*n* = 2). All operations achieved R0 resection successfully, with no intraoperative complication. Eighteen patients recovered without complications while three patients died from intra-abdominal hemorrhage due to a pancreatic fistula, though notably the bleeding was not at the arterial anastomosis site. All reconstructed arteries showed adequate patency at follow-up. The median postoperative survival was 11.6 months in all the 11 patients with pancreatic adenocarcinoma.

**Conclusion:**

Pancreatectomy combined with arterial resection and reconstruction is a feasible treatment option. The microsurgical technique is critically important to achieving a successful and patent arterial anastomosis.

## Background

Currently, surgical resection is considered the only possible curative approach for pancreatic tumors [1–3]. However, postsurgical outcomes remain poor as most patients present with advanced stage disease [4]. Due to their anatomy and biological properties, many of these tumors invade the surrounding tissues, including major vessels such as the hepatic artery (HA) and the superior mesenteric artery (SMA) [5]. Under these conditions, pancreatectomy combined with major arterial resection and reconstruction is the only method for R0 resection that can prolong postoperative survival. Some low-grade malignant tumors, such as solid-pseudopapillary tumor of the pancreas (SPT) and neuroendocrine neoplasm, may also invade the major vessels around the affected site. In these cases, R0 resection always requires arterial resection and reconstruction which often offers a better prognosis than patients with pancreatic adenocarcinoma.

Nevertheless, whether pancreatectomy should be combined with arterial resection and reconstruction in pancreatic tumors with major arterial involvement remains controversial [6]. Those opposed to this approach consider that arterial resection may not only add difficulty to the resection but can also influence the hepatic or intestinal arterial blood flow, thus increasing the postoperative morbidity and mortality. In contrast, some surgeons favor arterial resection because of the greater chance of achieving R0 resection in pancreatectomy [7–10]. In cases with major artery involvement, forcible separation of the tumor from the artery wall can cause arterial injury and even further spread of the tumor. One expert consensus statement has suggested that SMA margin is the most frequently positive during pancreatectomy [11], and R0 resection is crucial to extending patient survival [12]. If feasible, pancreatectomy combined with arterial resection and reconstruction may provide the only possibility of pancreatectomy with negative margins [4, 13].

Our center has a great deal of experience in living donor liver transplantation (LDLT) which requires microsurgical techniques. All hepatic arterial reconstructions are performed under an operating microscope with an extremely low rate of HA thrombosis [14]. Based on this experience, we conducted a total number of 21 pancreatectomies combined with arterial resection and reconstruction using similar techniques. The methods used in arterial reconstruction included direct anastomosis, arterial transposition, and arterial bypass with vascular graft. Herein, we share our experience and outcomes of artery resection and reconstruction in pancreatectomies.

## Methods

### Patients

Twenty-one patients who underwent arterial resection and reconstruction during a pancreatectomy from January 2010 to December 2015 at our center were enrolled in the study. Patients with retroperitoneal tumors involving the pancreas and cancer of the distal common bile duct (CBD) and the duodenal ampulla were also included. Arterial invasion was verified by preoperative computed tomography (CT) and intraoperative observation of all patients. A tumor was considered borderline resectable if it contacted the SMA and anatomical variants, such as accessory right hepatic artery (RHA), replaced RHA or if it involved the common hepatic artery without extension to the celiac trunk or hepatic artery bifurcation, thus allowing for safe and complete resection and reconstruction. The types of arterial reconstruction, operation time, blood loss, diagnosis, postoperative hospital stay, perioperative complications, patency of the reconstructed artery, and outcomes were assessed.

None of the 21 patients received neoadjuvant chemoradiotherapy. Preoperative informed consent was obtained from all patients. Allogeneic frozen iliac artery grafts were used following the guidelines of the Ethical Committee of our hospital, the regulations of the Organ Transplant Committee of Zhejiang Province, China and the Declaration of Helsinki.

### Operative technique

Standard pancreaticoduodenectomy (PD) or total pancreatectomy (TP) was performed as previously described. When arterial invasion was suspected or the artery could not be separated from the tumor, arterial resection and reconstruction were performed.

**Fig. 1:**
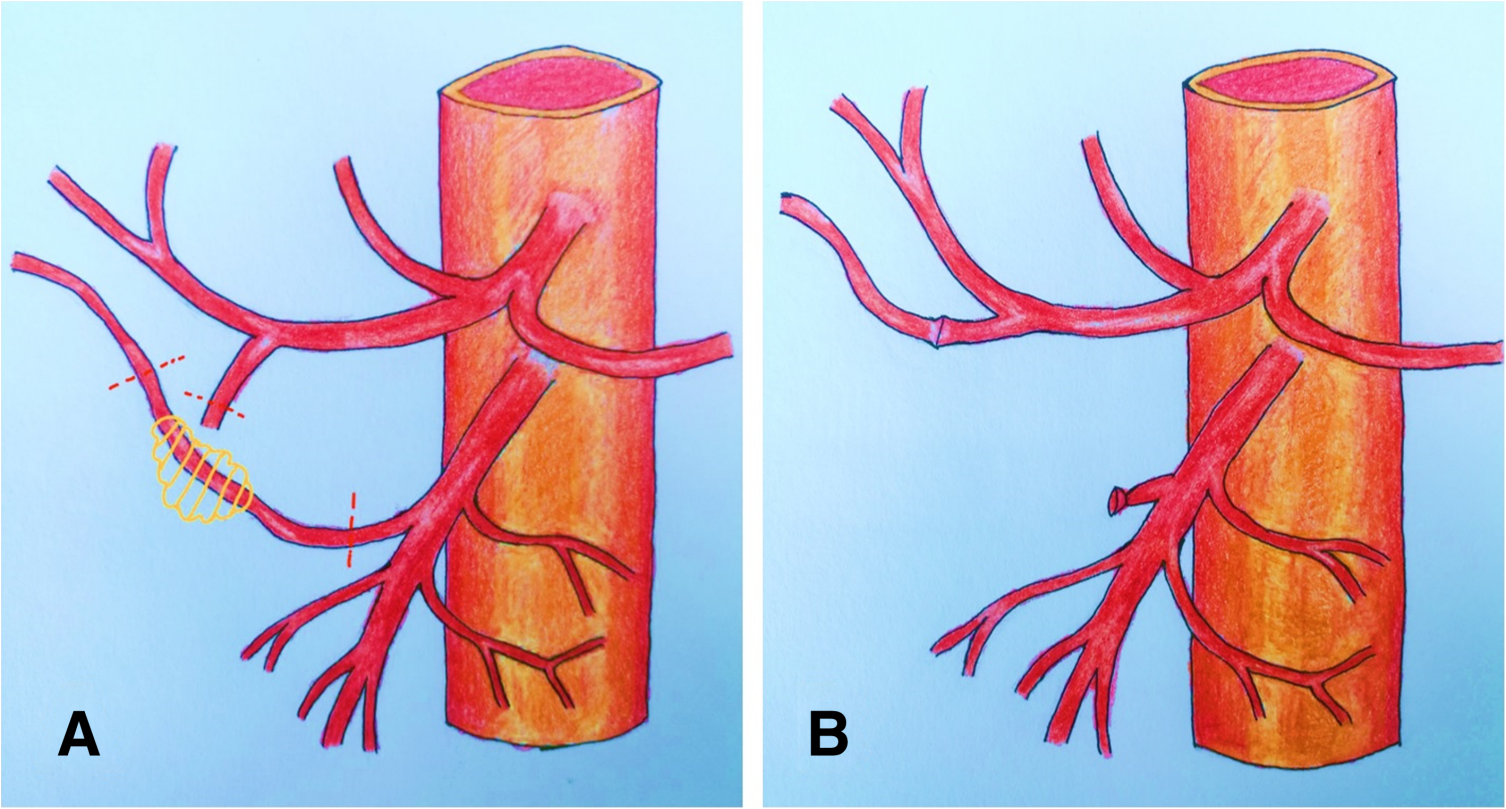
**a** Variant right hepatic artery (RHA) from the superior mesenteric artery with tumor invasion. **b** Gastroduodenal artery remnant used to reconstruct the variant RHA

**Fig. 2:**
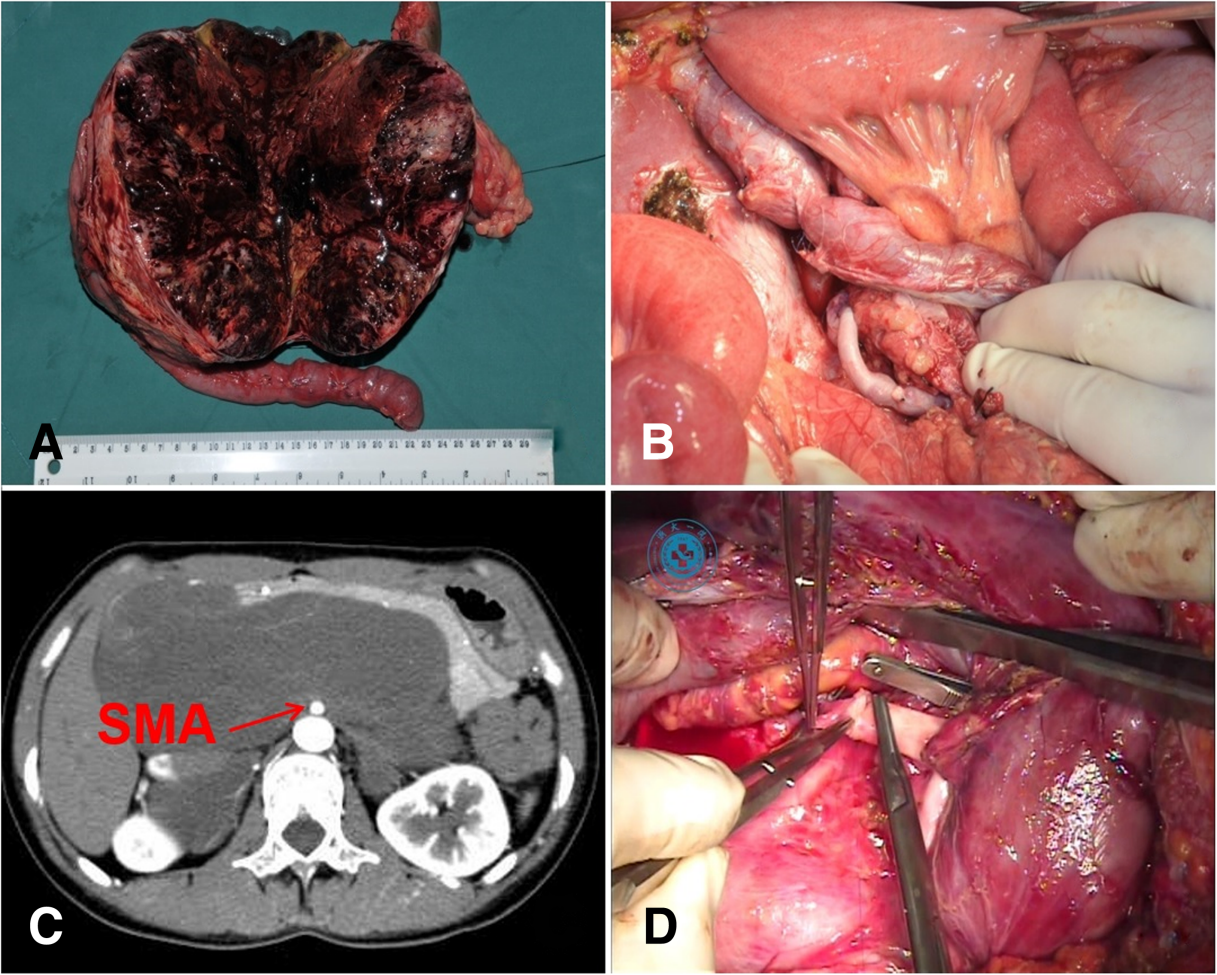
**a** A giant solid pseudopaillary tumor (16 cm) located in the pancreatic head and involving the superior mesenteric artery (SMA). **b** SMA reconstruction using an autologous great saphenous vein. **c** CT scan of a retroperitoneal ganglioneuroma involving the pancreatic head and SMA. **d** SMA reconstruction using a size-matched allogeneic frozen iliac artery graft

Generally, if the length of the resected arterial segment was less than 2 cm, direct end-to-end anastomosis was primarily considered; otherwise, arterial interposition was performed. When needed, the remnant gastroduodenal artery (GDA) from the PD resection was used as an autologous graft to reconstruct the variant RHA (Fig. 1). In the setting of a size mismatch the primary anastomosis could be perfomed in an end-to-side manner. However, arterial interposition was usually performed when none of the above methods were feasible. The autologous great saphenous vein (GSV) was the most preferred graft. In our study, size-matched allogeneic frozen iliac artery grafts were used in female patients whose GSVs were too small to match HA or SMA (Fig. 2). Arterial reconstruction was performed under an operating microscope.

**Fig. 3:**
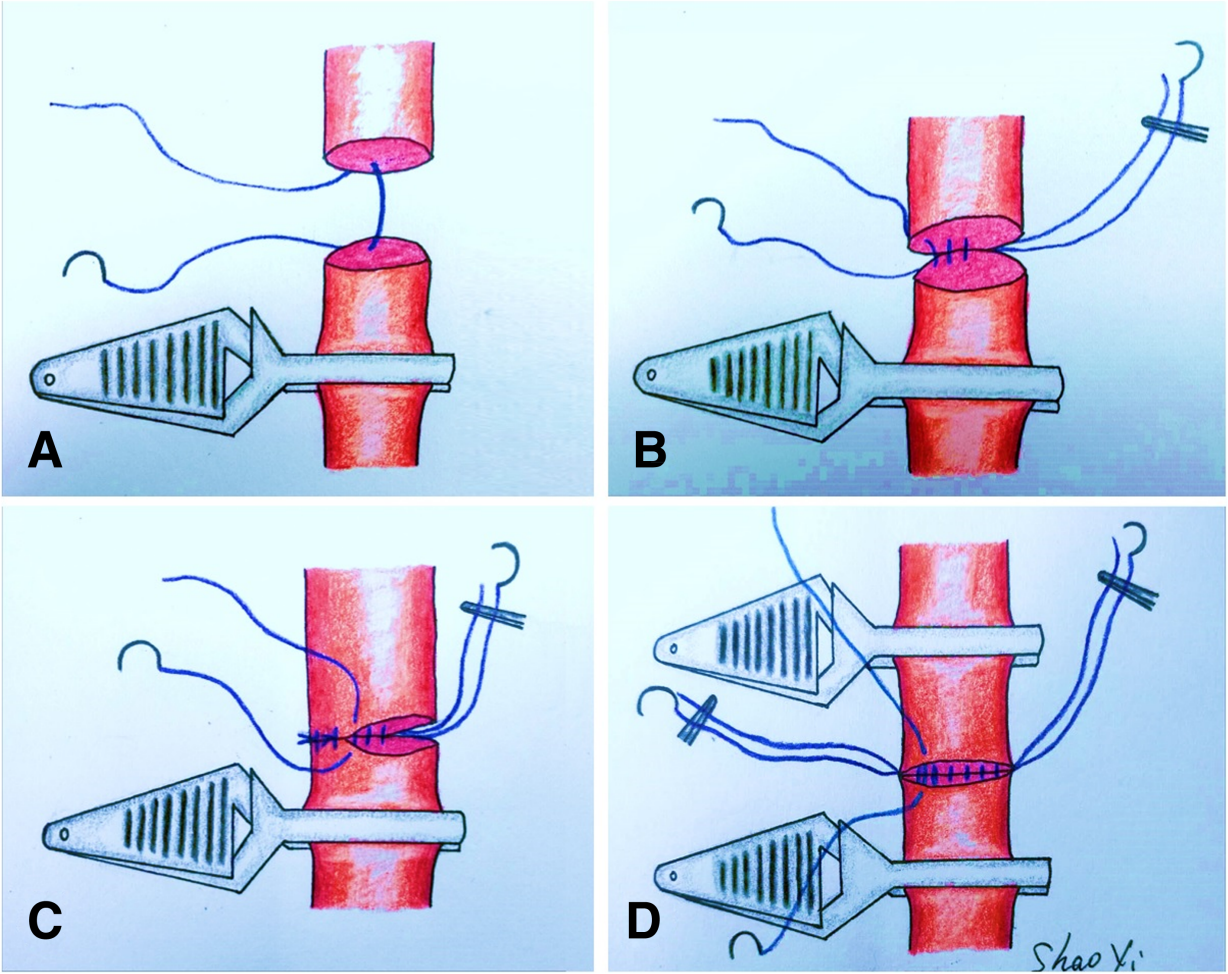
**a** A single microvascular clamp was placed on the proximal arterial stump leaving the distal artery free unclamped. **b** The anastomosis was started with a central stitch on the posterior wall. Subsequent the sutures were advanced counter-clockwise, adjacent to the previous suture. **c** The first central knot was pulled by the assistant to the left side to improve visualization of the anterior wall anastomosis. **d** The distal artery was clamped and the anterior wall was completed in a similar manner

A single microvascular clamp was placed on the proximal arterial stump after adequate arterial flow was confirmed. The distal artery remained free and unclamped, with backward flow detected from the portal network. The arteries were anastomosed in an end-to-end manner with interrupted sutures using 8–0 monofilament polypropylene suture (Surgipro II; Syneture, Norwalk, CT, USA), beginning with a central stitch on the posterior wall, knot tied on the outside surface. Next, the suture was advanced counterclockwise until the posterior wall was completed, with the assistant aiding visualization using both the central knot and following along each through. The distal artery was then clamped to prevent backflow, and counterclockwise suturing of the anterior wall was completed. The two penultimate stitch ends were left untied to aid the accurate insertion of the last stitch (Fig. 3). Frequent irrigation with heparinized saline solution (25 U/mL) was performed throughout the procedure to allow a clear visualization of the vascular wall. A temporary pause in ventilation was occasionally required to restrain movement during the transition of posterior to anterior wall suturing. Doppler ultrasonography was performed immediately after the completion of the arterial reconstruction. No anticoagulant or immunosuppressive drugs were used postoperatively.

### Follow-up

The blood flow of the reconstructed artery was assessed by postoperative Doppler ultrasonography at 1 week postoperation, as well as at 1, 6, and 12 months. CT angiography (CTA) was carried out after 6 months, one year, and every following year. Follow-up continued through August 31, 2018.

### Statistics

All data were analyzed using SPSS version 22.0 software (SPSS, Inc., Chicago, IL, USA). Categorical data were expressed as a percentage and compared using Fisher’s test. Continuous data were expressed as a median (range) and compared using unpaired t test. Calculations and graphs were made using GraphPad Prism 5.0. *P* < 0.05 was considered significant.

## Results

A total number of 9 men and 12 women, with a mean age of 54 years (range 19–78), were included in our study. The surgical procedures consisted of 19 PDs and 2 TPs. All procedures achieved successful R0 resection with no intraoperative complications. Operations were performed at the same institution by five surgeons each with over a decade of hepatopancreatobiliary experience. The arterial reconstruction was performed by the same microsurgical team that performed LDLT. The mean operation time was 589.2 min (range 187–855 min), and the mean time for arterial reconstruction was 25.1 min (range 21–33 min). The mean blood loss was 724 mL (range 200–2,000 mL). The mean tumor size was 4.9 cm (range 1.4–16 cm). The tumors were located at the pancreatic head (*n* = 10), the whole pancreas (*n* = 2), the distal CBD (*n* = 5), the ampulla (*n* = 2) and the retroperitoneum with pancreatic head involvement (*n* = 2). Fourteen patients had regional lymph node metastasis (Table 1).

**Table 1:** Patient Demographics

Age (year)	54 (19–78)
Sex	
Male	9
Female	12
Weight (Kg)	55 (38–70)
Height (cm)	164 (153–178)
Tumor diameter (cm)	4.9 (1.4–16.0)
Tumor location	
Pancreatic head	10
Distal pancreas	2
Total pancreas	2
Distal CBD	5
Duodenal ampulla	2
Pathology	
Adenocarcinoma	18
Moderate differentiation	15
Poor differentiation	3
Ganglioneuroma	2
SPN	1
Regional metastasis	
Yes	14
No	7
Distant metastasis	
Uncertain	4
No	17
Operational time (min)	589 (187–855)
Arterial reconstruction time (min)	25.1 (21–33)
Blood loss (mL)	723 (200–2000)
Post-operative hospital stay (day)	19 (8–34)

*CBD* common bile duct, *SPN* solid pseudopapillary neoplasm. Data are presented as “mean (range)”

Regional arterial resection and direct end-to-end anastomosis were used in 11 patients, whereas end-to-side anastomosis was performed in two cases. In one case, an accessory right hepatic artery (ARHA) was anastomosed to the common hepatic artery (CHA), in the second case, a replaced RHA from the SMA was anastomosed to the LHA (from the left gastric artery, LGA). In three cases, the RHA was anastomosed to the stump of the GDA. In one patient, the proper hepatic artery (PHA) was anastomosed to the LGA. Also, an allogeneic frozen iliac artery graft was interposed for the reconstruction of SMA in one case. In the remaining three cases with the SMA involvement of the tumor, autologous great saphenous vein (GSV) was used for SMA interposition. In one of those cases, the CHA was invaded simultaneously and was reconstructed in an end-to-end manner. Nine patients underwent portal vein resection additionally, with interpositional allogeneic frozen iliac artery grafts used in three cases due to inadequate length for a direct end-to-end anastomosis (Table 2).

**Table 2:** Surgical Outcomes

Case	Surgery	Invaded artery	Arterial reconstruction	Venous reconstruction	Arterial anatomy
1	PD	ARHA	ARHA-CHA	PV-SMV	ARHA from SMA
2	PD	RHA	RHA-GDA	No	LHA from LGA; RHA from SMA
3	PD	RHA	RHA-LHA	No	LHA from LGA; RHA from SMA
4	TP	CHA & PHA	PHA-CHA	SMV-SMV	Normal
5	PD	RHA	RHA-GDA	No	RHA from GDA
6	PD	CHA & PHA	PHA-CHA	SV + SMV- “Y” graft- PV	Normal
7	PD	CHA	CHA-CHA	No	SMA from Celiac axis, SA from aorta
8	TP	RHA	RHA-RHA	PV-PV	RHA from CHA
9	PD	PHA	PHA-LGA	SV + SMV- “Y” graft- PV	Normal
10	PD	CHA	CHA-CHA	No	Normal
11	PD	RHA	RHA-RHA	No	Normal
12	PD	PHA	PHA-CHA	PV-PV; SMV- “Y” graft-SMV	Normal
13	PD	PHA	PHA-CHA	No	Normal
14	PD	RHA	RHA-GDA	No	RHA from SMA
15	PD	CHA & PHA	PHA-CHA	PV-PV	Normal
16	PD	SMA	SMA-graft-SMA	No	SMA from Celiac axis; LGA from aorta
17	PD	CHA & PHA	PHA-CHA	PV-PV	Normal
18	PD	RHA	RHA-GDA	PV-PV	Normal
19	PD	SMA	SMA-GSV-SMA	No	Normal
20	PD	SMA	SMA-GSV-SMA	No	Normal
21	PD	CHA & SMA	CHA-CHA; SMA-GSVSMA	No	Normal

*PD* pancreaticoduodenectomy, *TP* total pancreatectom, *DP* distal pancreatectomy, *ARHA* accessory right hepatic artery, *CHA* common hepatic artery, *PHA* proper hepatic artery, *RHA* right hepatic artery, *LHA* left hepatic artery, *GDA* gastroduodenal artery, *LGA* left gastric artery, *SMA* superior mesenteric artery, *SA* splenic artery, *PV* portal vein, *SMV* superior mesenteric vein, *GSV* great saphenous vein. Graft: allogeneic frozen iliac vessel

Eighteen patients recovered and were eventually discharged after the operation. Their postoperative hospital stay was 19 ± 7 days without complications. Three patients died from intra-abdominal hemorrhage due to pancreatic fistulae resulting in sepsis during their hospital stay. It is worth mentioning that bleeding at the arterial anastomosis site was not found in any of the three patients, which was confirmed by digital subtraction angiography (DSA) or laparotomy. The mean follow-up duration was 16.7 months (0.3–71 months) (Table 3). Both the reconstructed arteries and veins showed adequate patency in the postoperative Doppler ultrasonography and subsequent CTA’s.

**Table 3:** Patient Outcomes and Disease Pathologies

Case	Sex	Age (year)	Location	Diameter (cm)	Pathology	Differentiation	Regional metastasis	Post-operative stay (day)	Complication	Outcome (months, cause of death)
1	M	52	Distal CBD	4	Adenocarcinoma	Moderately	Yes	19	None	Deceased (25.9, recurrence)
2	F	57	Distal CBD	1.5	Adenocarcinoma	Moderately	Yes	17	None	Deceased (27.5, recurrence)
3	F	57	Duodenal ampulla	2	Adenocarcinoma	Moderately	Yes	34	None	Deceased (9.7, recurrence)
4	M	49	Whole pancreas	6	Adenocarcinoma	Moderately	Yes	25	None	Deceased (12.9, recurrence)
5	M	69	Uncinate process	5	Adenocarcinoma	Poorly	Yes	20	None	Deceased (3.1, cerebral infarction)
6	F	67	Pancreatic head	4	Adenocarcinoma	Moderately	Yes	17	None	Deceased (10, recurrence)
7	M	65	Distal CBD	4.5	Adenocarcinoma	Moderately	Yes	–	Bleeding; sepsis	Deceased (0.3, bleeding; infection)
8	F	47	Whole pancreas	3	Adenocarcinoma	Moderately	Yes	34	None	Deceased (1.8, chemotherapy related)
9	M	49	Pancreatic head	4	Adenocarcinoma	Moderately	Yes	14	None	Deceased (37.7, recurrence)
10	M	67	Distal CBD	2	Adenocarcinoma	Moderately	Yes	22	None	Lost (0.7)
11	F	54	Duodenal ampulla	2	Adenocarcinoma	Poorly	Yes	11	None	Deceased (13.4, recurrence)
12	F	53	Pancreatic head	4	Adenocarcinoma	Moderately	Yes	12	None	Deceased (11.6, recurrence)
13	M	78	Pancreatic head	3	Adenocarcinoma	Moderately	No	20	None	Deceased (10, gastroplegia)
14	F	56	Pancreatic head	3	Adenocarcinoma	Moderately	Yes	22	None	Deceased (17, recurrence)
15	M	75	Pancreatic head	4	Adenocarcinoma	Poorly	No	–	Bleeding; sepsis	Deceased (0.6, bleeding; infection)
16	M	55	Distal CBD	1.4	Adenocarcinoma	Moderately	No	–	Bleeding; sepsis	Deceased (0.4, bleeding; infection)
17	F	60	Uncinate process	4	Adenocarcinoma	Moderately	Yes	15	None	Deceased (12.5, recurrence)
18	F	30	Uncinate process	4	Adenocarcinoma	Moderately	No	16	None	Deceased (11.8, recurrence)
19	F	49	Retroperitoneum with pancreatic head involvement	11	Ganglioneuroma	–	No	21	Pancreatic fistula (A)	Alive (60.6)
20	F	19	Pancreatic head	16	SPT	–	No	27	None	Deceased (11.6, recurrence)
21	F	23	Retroperitoneum with pancreatic head involvement	15	Ganglioneuroma	–	No	13	None	Alive (71)

*M* male, *F* female, *CBD* common bile duct, *SPT* solid pseudopapillary tumor

**Fig. 4:**
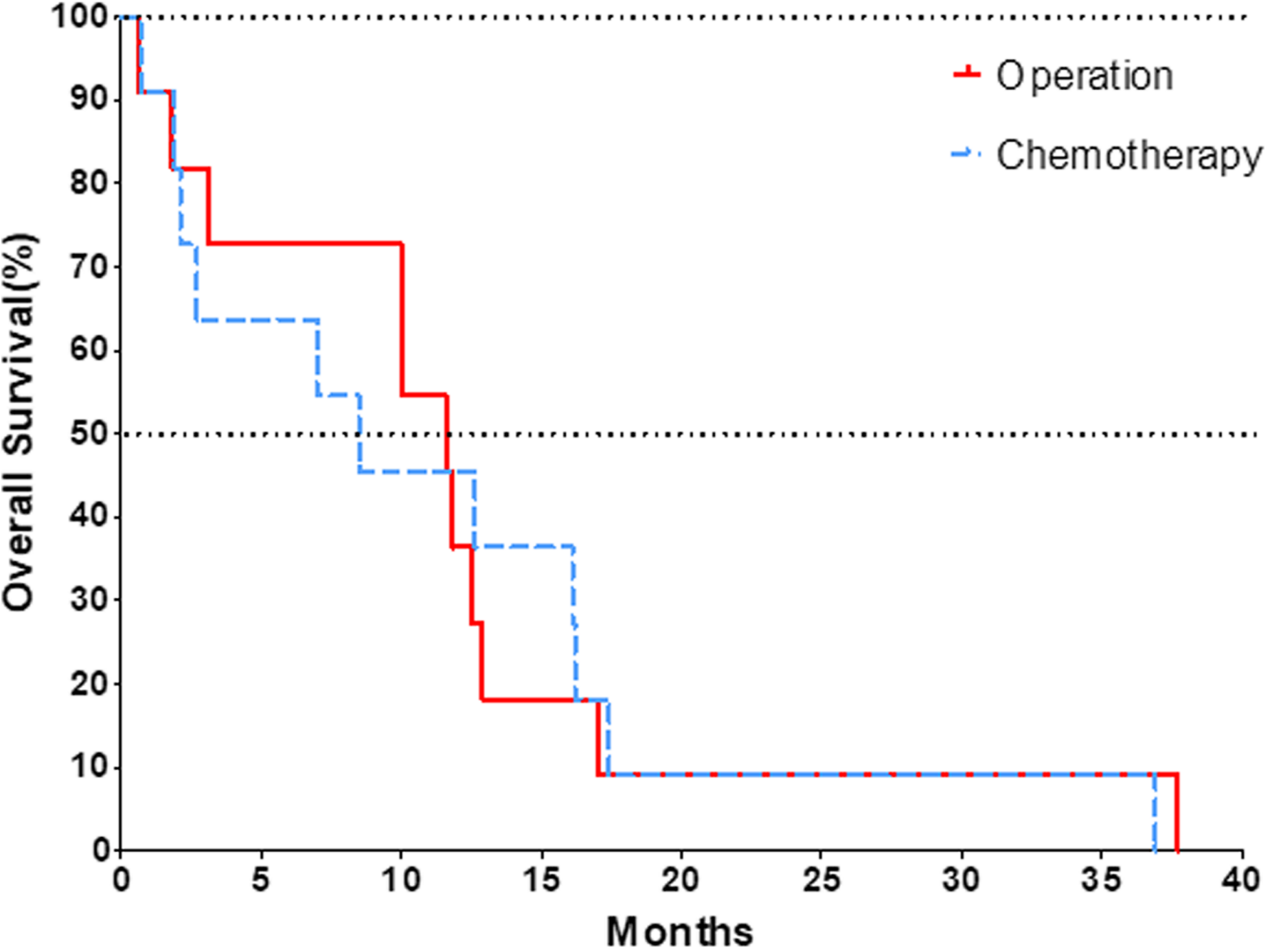
The median survival time was 11.6 months in operative group vs. 8.5 months in the chemotherapy group, *p* > 0.05

Eleven patients with pancreatic adenocarcinoma who received surgery were compared with eleven patients who received chemotherapy alone from January 2012 to August 2018 (Table 4 and Fig. 4). The oncologic outcomes were similar between the two groups with the median survival of 11.6 months in the operative group vs. 8.5 months in the chemotherapy group, *p* > 0.05.

**Table 4:** Outcomes of Patient with Pancreatic Adenocarcinoma

	Operative Group	Chemotherapy Group	*P* value
Total Patients	11	11	
Age, mean (yr)	58 (30–78)	61 (46–81)	0.5004
Sex			1.000
Male	5 (45.6%)	6 (54.4%)	
Female	6 (54.4%)	5 (45.6%)	
Tumor diameter, mean (cm)	4 (3–6)	4.1 (2.5–6)	0.7399
TNM stage	III	III	
Tumor location			0.0841
Pancreatic head	8 (72.7%)	6 (54.4%)	
Distal pancreas	1 (9.1%)	5 (45.6%)	
Total pancreas	2 (18.2%)	0 (0%)	
Chemotherapy protocols			0.0292
GEM	7 (63.6%)	2 (18.2%)	
GEMOX	0 (0%)	2 (18.2%)	
GS	1 (9.1%)	5 (45.6%)	
mFOLFIRINOX	0 (0%)	1 (9.1%)	
AG	0 (0%)	1 (9.1%)	
None	3 (27.3%)	0 (0%)	
Median Survival (mo)	11.7 (0.6–37.7)	11.1 (0.7–36.9)	0.922

*GEM* gemcitabine 1000 mg/m^2^ days 1, 8, 15 every 4 wks; GEMOX: gemcitabine 1000 mg/m^2^ days 1, 8, 15 + capecitabine 1660 mg/m^2^ days 1–21 every 4 wks; GS: gemcitabine 1000 mg/m^2^ days 1, 8+ tegafur gimeracil oteracil potassium 80-120 mg days 1–14 every 3 wks, mFOLFIRINOX: day 1 oxaliplatin 68 mg/m^2^ + calcium folinate 400 mg/m^2^ + irinatecan 135 mg/m^2^ + 5-fluorouracil 2400 mg/m^2^ every 2 wks; AG: gemcitabine 1000 mg/m^2^ + albumin-bound paclitaxel regimens 125 mg/m^2^ days 1, 8, 15 every 4 wks

## Discussion

Fortner et al. reported no significant improvement in the postoperative outcomes for arterial resection and reconstruction in pancreatectomy [15], and pancreatic cancer with arterial involvement is considered a contraindication to resection by most surgeons. However, limited studies have reported more promising outcomes [16–19]. For instance, Karyn et al. reviewed twelve cases with pancreatic cancer that underwent a pancreatectomy with hepatic artery or celiac artery resection and established a median survival of 17 months and a 3-year survival of 17% [20]. Moreover, Hodaka et al. concluded that patients with the possibility of R0 surgery might benefit from arterial resection and reconstruction. In their study, the median survival times for R0 and R1/R2 resections were thirteen and six months, respectively [21]. Additionally, Mollberg et al. analyzed five investigations with comparative data and found significant improvements in the 1-year (OR: 4.28; *P* < 0.0001) and 2-year (OR: 19.65; *P* < 0.0001) survival in patients with arterial resection compared with patients without [22]. Even though pancreatic cancer resections are associated with high mortality and morbidity, pancreatectomy combined with arterial resection may prolong the survival in select patients.

The pancreas, though retroperitoneal, lies adjacent to major vessels such as the celiac artery, HA and SMA. Furthermore, HA variants have been reported in as many as 20 to 50% of patients [23]. Hiatt et al. reported 10.6% replaced or accessory RHA originating from the SMA. Especially in this setting of arterial variants, major vessel invasion by the pancreatic tumor can be found in nearly 45% [24]. Moreover, lymph node metastases occur frequently even in patients with early-stage pancreatic cancer [25]. This makes resection of the involved artery necessary for radical lymphadenectomy and en-bloc resection.

In our series, the diameters of the HA and SMA were 2–3 mm and 6–8 mm, respectively. Thinner arterial walls secondary to tumor invasion, increases the risk for arterial thrombosis, anastomotic bleeding, and stenosis after arterial reconstruction. The incidence of the above complications affects the blood flow to the liver and intestines, which can lead to hepatic failure or intestinal ischemia. Many studies have concluded that microsurgical techniques can considerably decrease the risk of hepatic arterial thrombosis after LDLT [26]. The HA reconstruction technique can also be applied to the arterial reconstruction in pancreatoduodenectomy. Consequently, this microsurgical technique was important for achieving a safe and effective arterial anastomosis.

In the present study, twenty-one patients who underwent pancreatectomy with resection and reconstruction of a major artery were reviewed. No intraoperative complications were observed in any of these cases. Although three patients died in the hospital due to intra-abdominal hemorrhage and sepsis after the operation, none of these complications were attributed to the arterial reconstruction given DSA and exploratory laparotomy results.

Two critical points were the length and diameter of the involved arteries. We have previously observed that direct end-to-end anastomosis is feasible when the length of the resected arterial segment is less than 2 cm (data not shown). Otherwise, arterial transposition or arterial bypass was a better option. The choice of the proximal artery for the arterial transposition is diverse. Theoretically, the stump of the HA or SMA can be anastomosed with any of the proximal arteries, provided their lengths and diameters were suitable. The GDA is routinely resected in PD, and its stump may be an appropriate option to reconstruct the RHA [27, 28], especially in cases where the RHA arises from the SMA. Some surgeons have also used the splenic artery (SA) transposition to reconstruct the HA or SMA [29–31]. However, there is the potential for SA dissociation and an added risk of splenic due to the multiple SA branches. Hence, the SA is seldom utilized for the HA or SMA reconstruction in pancreatectomies.

Additionally, vascular bypass offers an important option for arterial reconstruction [32]. GSV has been widely used in coronary artery bypass grafting. With advantages such as increased success rate and sufficient length, the GSV has primarily been considered the graft of choice for the arterial reconstruction in pancreatectomy. Allogeneic vessel graft is another alternatives for revascularization. At our center, frozen allogeneic iliac arteries are frequently utilized for venous reconstruction (segment V/VIII) of the liver graft in LDLT. Based on this experience, we used an allogeneic frozen iliac artery in one case and autologous GSV in three cases for the SMA reconstruction. Just as there was a wide range of variations to these methods, surgeons should determine the best choice based on the peculiarities of each patient situation.

As the vascular reconstruction procedure is time-consuming, surgeons should be cautious of the artery occlusion time. Bin-Li et al. reported a HA- and SMA-occlusion time during revascularization of 38–50 min and 35–50 min, respectively. No patients who underwent PD with arterial reconstruction experienced liver or small bowel ischemia [17]. In the present study, the arterial anastomosis took 25.1 min (21–33 min) to complete, without any complications. When the PV and HA needed to be reconstructed simultaneously, the SMA was always temporarily occluded to prevent congestion of the small intestine if HA-occlusion exceeded one hour.

Multimodality treatment approaches have become routine for pancreatic cancer therapy [33]. Neoadjuvant chemotherapy, which may be superior at improving the R0 resection rate and assessment of the sensitivity to chemotherapeutic drugs, has attracted substantial attention [34, 35]. Our series was conducted from 2010 to 2015. Gemcitabine was the first line therapy for neoadjuvant chemotherapy for locally advanced pancreatic cancer during that time, however, its ORR was unsatisfactory compared with FOLFIRINOX or gemcitabine+albumin-bound paclitaxel regimens. Secondly, most patients in China diagnosed with advanced pancreatic cancer prefer radical resection due to the financial burden associated with the chemotherapeutic regimen. One limitation of this study is the relatively small sample size. More patients are required to expand the research foundation and our understanding in this field.

## Conclusion

Pancreatectomy combined with arterial resection and reconstruction is a feasible treatment option, and can be considered for select patients. Microsurgical technique is critically important for a successful and patent arterial anastomosis.

## Data Availability

The data that support the findings of this study are available from Division of Hepatobiliary and Pancreatic Surgery, Department of Surgery, however, restrictions apply to the availability of this data; which was used under license for the current study, therefore are not publicly available. Data are however available from the authors upon reasonable request, with permission from the Division of Hepatobiliary and Pancreatic Surgery, Department of Surgery.

## Abbreviations

ARHA: Accessory right hepatic artery
CBD: Common bile duct
CHA: Common hepatic artery
CT: Computed tomography
CTA: CT angiography
DSA: Digital subtraction angiography
GDA: Gastroduodenal artery
GSV: Great saphenous vein
HA: Hepatic artery
LDLT: Living donor liver transplantation
LGA: Left gastric artery
LHA: Left hepatic artery
PD: Pancreaticoduodenectomy
PHA: Proper hepatic artery
RHA: Right hepatic artery
SMA: Superior mesenteric artery
SPT: Solid-pseudopapillary tumor of the pancreas
TP: Total pancreatectomy
